# The Home Literacy Environment Is a Correlate, but Perhaps Not a Cause, of Variations in Children’s Language and Literacy Development

**DOI:** 10.1080/10888438.2017.1346660

**Published:** 2017-07-25

**Authors:** Marina L. Puglisi, Charles Hulme, Lorna G. Hamilton, Margaret J. Snowling

**Affiliations:** a University of Oxford, Federal University of São Paulo; b University College London; c York St John University; d University of Oxford, St. John’s College

## Abstract

The home literacy environment is a well-established predictor of children’s language and literacy development. We investigated whether formal, informal, and indirect measures of the home literacy environment predict children’s reading and language skills once maternal language abilities are taken into account. Data come from a longitudinal study of children at high risk of dyslexia (*N* = 251) followed from preschool years. Latent factors describing maternal language were significant predictors of storybook exposure but not of direct literacy instruction. Maternal language and phonological skills respectively predicted children’s language and reading/spelling skills. However, after accounting for variations in maternal language, storybook exposure was not a significant predictor of children’s outcomes. In contrast, direct literacy instruction remained a predictor of children’s reading/spelling skills. We argue that the relationship between early informal home literacy activities and children’s language and reading skills is largely accounted for by maternal skills and may reflect genetic influences.

It is well established that the home literacy environment is an important predictor of children’s language and literacy development (Frijters, Barron, & Brunello, 2000; Levy, Gong, Hessels, Evans, & Jared, 2006; Senechal & Lefevre, 2002). The “home literacy environment” usually refers to activities undertaken by family members at home that relate to literacy learning (Bracken & Fischel, 2008; Burgess, Hecht, & Lonigan, 2002; Payne, Whitehurst, & Angell, 1994; Rashid, Morris, & Sevcik, 2005) as well as the literacy resources in the home and parental attitudes toward literacy (Martini & Sénéchal, 2012; Weigel, Martin, & Bennett, 2006). It is usually measured by rating scales and can be divided into “formal” and “informal” home-based literacy interactions (Senechal & Lefevre, 2002).

Informal literacy interactions include a variety of activities in which parents read to their child or direct their attention to print in the environment, such as advertisements or street names; such daily shared reading experiences between parents and children are estimated to add up to approximately 1,000 hr of print exposure at the beginning of kindergarten (Adams, 1990). Formal literacy interactions, on the other hand, refer to activities in which adults directly teach reading or promote print-related skills at home (e.g., writing the child’s name, teaching letter names and sounds). Whereas formal literacy interactions in the preschool years are generally associated with better “code-based” literacy skills, including decoding, at or around school entry (Foy & Mann, 2003; Manolitsis, Georgiou, & Tziraki, 2013; Martini & Sénéchal, 2012), informal literacy practices (most commonly shared book-reading in the home) appear to be more closely associated with the development of broad oral language skills, including vocabulary knowledge, and thus indirectly with reading comprehension later in development (Hamilton, Hayiou-Thomas, Hulme, & Snowling, 2016; Sénéchal, 2006).

However, the separation of informal and formal literacy activities is not clear-cut, and the balance of these activities may change over time. There is evidence, for example, that parents adjust the frequency with which they teach their young children about letters and words as a function of their child’s progress, increasing support where progress is slower than expected (Sénéchal & Lefevre, 2014). Furthermore, once young children have begun to acquire the alphabetic principle, shared storybook reading can provide a facilitative context for the development of code-based emergent literacy skills (Mol, Bus, & de Jong, 2009). Together these sorts of findings may explain inconsistencies in the literature, which reports both positive (Evans, Shaw, & Bell, 2000; Hood, Conlon, & Andrews, 2008; Senechal & Lefevre, 2002) and negative (Sénéchal & Lefevre, 2014; Silinskas et al., 2012) associations between parental teaching and children’s early literacy skills.

There are also indirect (or passive) influences of the home literacy environment on children’s attainments; indeed, literate parents provide good role models for family members who are learning to read (Saracho, 1997). Passive influences are usually assessed by measures of parents’ literacy orientation or by the frequency of literary activities they take part in, and it is widely believed that the homes of more literate parents provide richer opportunities for literacy than those of parents with low levels of language and literacy (Phillips & Lonigan, 2005). Studies that have directly compared active and passive home literacy activities have found that the active components (i.e., parent–child interactions) are better predictors of children’s skills than the passive measures (Bracken & Fischel, 2008; Burgess et al., 2002). However, in a sample of children with reading disorders, Rashid and colleagues (2005) found the opposite pattern: Passive—but not active—home literacy experiences accounted for a significant amount of variance in children’s reading comprehension and spelling after controlling for child IQ and maternal education.

The relationship between the home literacy environment and children’s language and literacy skills may also differ in families of children with and without language impairment (Skibbe, Justice, Zucker, & McGinty, 2008). For example, in a study of 251 kindergarten children with language impairment, Petrill, Logan, Sawyer, and Justice (2014) reported nonlinear concurrent associations between the frequency of shared reading and emergent literacy skills. Parents of children with language difficulties may also adjust their linguistic input during home literacy activities in line with their children’s productive skills (Majorano & Lavelli, 2014). In contrast, studies to date have reported minimal differences in the frequency and quality of literacy activities provided by parents who are dyslexic readers in comparison with typical readers (Elbro, Borstrøm, & Petersen, 1998; Laakso, Poikkeus, & Lyytinen, 1999; Van Bergen, Jong, Plakas, Maassen, & Leij, 2011), and the limited literature has not taken into account the reading skills of parents themselves.

Genetic factors also have an important influence on literacy development. The correlation between the home literacy environment and literacy development likely reflects genetic as well as environmental influences because biologically related family members in the same household share both genes and aspects of the environment (Hart et al., 2009). Gene–environment (*ge*) correlation refers to the influence of parental genes working through the environment (Rutter, Moffitt, & Caspi, 2006). A passive *ge* correlation is observed when there is a correlation between the parent’s genotype and both the child’s genotype and their environment. Thus, parents who have a genetic predisposition to read well not only pass this predisposition on to their children (genetically) but also are likely to provide their children with a better environment for learning to read. An evocative *ge* correlation refers to the association between an individual’s genetically influenced behaviour and others’ reactions to that behaviour; children who have a genetic predisposition to read well may evoke in others a desire to read with them, leading to greater engagement with reading. An active *ge* correlation is observed when there is an association between a given genetic endowment and the environmental niches that individual selects such that children who have a genetic predisposition to read well seek out reading opportunities more often than those who struggle to read.

To disentangle genetic and environmental effects on children’s academic achievement requires data from genetically sensitive designs. A meta-analysis of genetically sensitive studies estimated the heritability of reading to be .73 and spelling .64, whereas shared environmental influences accounted for only 10% of the variance in reading (de Zeeuw, de Geus, & Boomsma, 2015). Similarly, a review by Olson, Keenan, Byrne, and Samuelsson (2014) concluded that although environmental influences are in general statistically significant for reading disability, the average influence of genes is about twice as strong as the shared environmental influence.

Van Bergen, van Zuijen, Bishop, and de Jong (2016) presented evidence for a possible role of genetic factors in explaining the correlation between the home literacy environment and the reading skills of children 6–17 years of age who had completed at least 14 months of formal reading instruction. As well as measuring the home literacy environment with three types of measure (how long parents spent reading, subscriptions to magazines and newspapers, estimates of the number of books in the home), they assessed parents’ reading fluency and level of education. Parental reading fluency was a moderate predictor of children’s reading fluency (*r*s = .29–.32), and after controlling for this variable, the only other measure that predicted children’s reading fluency was the estimated number of books in the home. The authors argued that the correlation between some aspects of the home literacy environment and child reading may be the product of genetic effects but that the number of books in the home may represent a true environmental effect. However, it is notable that this study did not have any direct measures of the literacy activities parents undertook with their children, which is how the home literacy environment is typically defined. In a similar vein, Rashid et al. (2005) found that the home literacy activities undertaken by parents of children with reading disabilities were not significantly related to their academic outcomes once parents’ own literacy activities at home were taken into account. Children’s age range in this sample (6;6–8;6) was narrower than in Van Bergen et al. (2016), but participants were all attending elementary school at the time of screening (Grade 1 or 2).

Together these findings from measures of parental literacy skills suggest a genetically mediated explanation for the correlation between the home literacy environment and children’s literacy skills is plausible. In other words the correlation between the home literacy environment and children’s literacy skills may, at least in part, reflect the genetic relationship that parents have with their children, rather than being a purely environmental influence. To investigate this issue further, the current study assessed the role of the home literacy environment in predicting child language and literacy outcomes and then considered its influence when maternal literacy skills were controlled.

We know that different aspects of home literacy practices predict children’s oral language and emergent literacy skills in a subsample of the current cohort comprising only children at family risk of dyslexia and controls and not including children with language impairment (Hamilton et al., 2016). Our goal here was to examine the relationship between the home literacy environment and child reading/spelling and language outcomes in a larger sample while taking into account the effects of objectively measured maternal language and phonological skills. We assessed five components of the home literacy environment: number of children’s books, frequency of shared reading, maternal familiarity with children’s books, maternal familiarity with adult fiction, and direct literacy instruction. We expected direct literacy instruction to predict reading/spelling skills (Martini & Sénéchal, 2012) and the other informal measures, which we labelled storybook exposure, to have broader associations with measures of both children’s language and reading/spelling skills (e.g., Bus, van Ijzendoorn, & Pellegrini, 1995).

To assess the possible role of genetic influences on child language and literacy skills, we included separate measures of maternal language and phonological skills. Although the heritability of general language skills has been estimated to be 64% (de Zeeuw et al., 2015), other behaviour-genetic studies indicate that phonological processing skills, such as the ability to repeat nonwords, are more highly heritable and less susceptible to environmental effects than broader language abilities, such as vocabulary (Hayiou-Thomas, 2008). This leads to the prediction of a stronger association between maternal phonology and child reading/spelling than between broader (nonphonological) measures of mother’s language and child language and literacy outcomes.

In summary, the study was designed to test the following hypotheses:

1. Measures of maternal language skills will predict the home literacy environment that mothers provide as defined by the content and frequency of literacy interactions.
2. Measures of the home literacy environment will predict children’s language and reading/spelling skills. Based on previous research, we expected storybook exposure to be a moderate correlate of children’s outcomes, predicting both general language and emergent literacy skills (Bus et al., 1995). In contrast, we expected direct literacy instruction to be a stronger predictor of children’s reading/spelling skills (Martini & Sénéchal, 2012).
3. A final, critical question for this study is to identify the extent to which measures of the home literacy environment will predict children’s outcomes after controlling for variations in mothers’ language and phonological skills. If these effects are really genetic effects (cf. Van Bergen et al., 2016), controlling for maternal skills may eliminate effects of the home literacy environment on children’s language and reading/spelling skills.

## Method

### Ethical considerations

Data are reported from the first three phases of the Wellcome Language and Reading Project, a longitudinal study of children at high risk of dyslexia. Ethical clearance for the study was provided by the University of York, Department of Psychology Research Ethics Committee and the National Health Service Research Ethics Committee. Parents provided informed consent for their child to be involved.

### Participants

Families were recruited to the study via advertisements and speech and language therapy services (for details, see Nash, Hulme, Gooch, & Snowling, 2013). The sample of the Wellcome Project (*N* = 260) overrepresented children who were at cognitive risk of later reading problems. The proportion of children who met criteria for language impairment at the beginning of the study was also considerably higher than the prevalence of this disorder in the population (see Nash et al., 2013, for details). The sample represented a broad range of socioeconomic backgrounds, but people of high socioeconomic status (SES) were overrepresented (49.5% of mothers had more than 21 years of education, the highest category represented).

Children with previous diagnoses of chronic illness, deafness, neurological, or psychiatric disorders and children speaking English as a second language were excluded from the sample. Only participants who were assessed during the first 3 years of the project and were up to 4½ years of age at the first assessment were included in the analysis. Nine sibling pairs were in the sample; one from each pair was excluded at random, leaving 251 children whose data are reported here (59.4% male).

### Research design

In this study we assess whether the home literacy environment predicts children’s language and literacy outcomes after controlling for variations in maternal language and phonological skills. To be able to demonstrate clear predictive effects, we opted to test each of the three main constructs (maternal skills, home literacy environment, and children skills) at different time points.

At Time 1, when children were on average 3½ years old, we assessed maternal language and phonological abilities because these skills are recognized as tapping individual differences which underpin literacy (Olson et al., 2011). The measures were the first predictors in the model and were crucial for our research design because they addressed whether the variance in children’s skills explained by the home literacy environment could be accounted for by maternal abilities. We included data from mothers in the analyses because in all but one case the mother was the primary caregiver and hence likely to have a strong influence on the home literacy environment; in addition, there was a great deal of missing data for fathers.

At Time 2, 1 year later (children’s age averaged 4½ years), we focused on exploring different aspects of the home literacy environment through questionnaires completed by the main caregiver in the context of an interview. In the models that follow, this construct was predicted by maternal skills and in turn was a predictor of the main outcome—children’s skills.

When children were 5½ years old (Time 3) we assessed their language, reading, and spelling skills. These measures represented the main outcomes of interest and tapped children’s performance when they were in their 1st year of formal education (Reception class of primary school in England).

Time 1 and 2 data were collected at the participants’ homes using assessments and questionnaires. At Time 2, the children had been in school for 2.68 months on average. Time 3 data were collected in the children’s schools.

### Tests and procedures

The data set from which the current measures were drawn is large; we chose measures that reflect the main constructs used in previous studies of the home literacy environment and literacy development and that had good distributions of scores. For all measures of maternal and child skills, and for storybook exposure, we defined latent variables for the purpose of modelling the data by combining measures tapping different aspects of the same construct.

### Maternal skills (time 1)

#### Maternal language

##### Vocabulary

Mothers completed the Vocabulary subtest from the Wechsler Abbreviated Scale of Intelligence (Wechsler, 1999) in which a series of words are defined up to a discontinuation rule. Responses are scored 0, 1, or 2 using the criteria in the manual for a maximum of 42 items. The reported split-half reliability is .96. Raw scores were converted into standard scores for the analysis.

##### Grammar

Grammatical skills were assessed using a sentence-reordering subtask adapted from the Test of Adolescent and Adult Language (Hammill, Brown, Larsen, & Wiederholt, 2007). The maximum raw score was 20 (α = .79).

##### Oral language competence

Parents completed the Communication Checklist–Adult (Whitehouse & Bishop, 2009), which taps language functioning in a series of oral language domains, including phonology, vocabulary, syntax, and pragmatics (reported α for all domains > .90). There are 70 behavioral statements and informants (here it was self-report) must judge whether the individual in question demonstrates that behavior: less than once a week or never (a score of 0), at least once a week but not every day (1), once or twice a day (2), or several times a day or always (3). We used the *z* scores of the total oral language measure and transformed outliers at the lower edge of distribution (*n* = 7) to the lowest score minus 0.1 *z* score.

#### Maternal phonology

##### Nonword repetition

Mothers were asked to repeat 28 nonwords of three to five syllables taken from the Nonword Memory Test (NWRep1; α = .70; Gathercole & Baddeley, 1996) and five complex multisyllabic nonwords from the Comprehensive Test of Phonological Processing (NWRep2; α* = *.21; Wagner, Torgesen, & Rashotte, 1999). Items from the NWRep2 were included to guard against ceiling effects on NWRep1.

##### Phonological awareness

A Spoonerism task was used to assess phonological awareness (α = .76). Participants heard 12 pairs of words and were required to switch the initial phonemes between the two words and give a verbal response. The measure used was the mean time (in seconds) taken to complete a trial accurately. This variable was log-transformed to correct for a right-skewed distribution.

### Home literacy environment (time 2)

Measures of the home literacy environment were collected during a semistructured interview and via checklists at a home visit at Time 2. These measures assessed informal activities related to storybook exposure (number of children’s books, frequency of shared reading, maternal familiarity with children’s books, maternal familiarity with adult fiction) and formal activities (direct literacy instruction).

#### Storybook exposure

A measure of storybook exposure was taken as a proxy for the amount of opportunity the child had to experience storybook reading.

##### Number of children’s books

Parents were asked to estimate the number of children’s books in the home on a 7-point scale (cat1: 0–20; cat2: 21–40; cat3: 41–60; cat4: 61–100; cat5: 101–150; cat6: 151–200; cat7: >200). Test–retest reliability after a 4-week interval was .78.

##### Frequency of shared reading

Primary caregivers were asked to report how often they read storybooks to their children in a typical week (summed responses to two items: How many times in a typical week do you read a bedtime story with your child? How many times in a typical week do you read stories with your child at other times of day?). The final outcome corresponded to the sum of these frequency questions. Test–retest reliability after a 4-week interval was .88.

##### Maternal familiarity with children’s books

This variable was measured using two parent checklist measures (Hamilton et al., 2016): (a) the Child Title Checklist containing 30 titles of popular children’s books intermixed with 30 plausible foils. The selected titles were the most frequently occurring items from online bestseller lists and lists of most frequently borrowed picture books provided by local librarians. None of the selected titles had been filmed or televised at the time of data collection, and all were available in public libraries; (b) the Child Author Checklist containing 40 authors associated with the titles elicited for the Child Title Checklist and 40 foils. The primary caregiver was asked to check the box next to every title/author they recognised. For each checklist, raw scores were calculated by subtracting the number of foils checked from the number of target items checked to correct for guessing. Following previous research (e.g. Stainthorp, 1997) it is assumed that parents who engage in more shared reading with their children will recognise a greater number of children’s book titles and titles and authors. The final outcome was the mean composite of the *z*-scores of the two checklists (α = .89).

##### Maternal familiarity with adult fiction

This variable was assessed by the Adult Author Checklist, which consists of 40 authors of contemporary fiction, representing a broad range of genres, and 40 foils (Acheson, Wells, & Macdonald, 2008; Stanovich & West, 1989). Maternal familiarity with adult fiction was considered to be an indirect measure of storybook exposure, as we would expect that parents who read for pleasure more also value reading books with their children more. *Z* scores were used in the analysis (α = .93). Negative scores on the checklists (Child Title Checklist, Child Author Checklist, and Adult Author Checklist) suggest that the respondent has guessed the answers and were therefore rescored as zero.

#### Direct literacy instruction

This variable was a mean composite of the *z* scores of three items from a family interview (α = .60), adapted from Sénéchal and Lefevre (2002) as described in Hamilton et al. (2016). Parents were asked to rate how often they taught their children to recognise letters, read words, and write words, using a 5-point scale: 1 (*never/occasionally*), 2 (*about once a month*), 3 (*about once a week*), 4 (*several times a week*), and 5 (*daily*). This measure was used as an observed variable in our models.

### Children’s language, phonological and literacy skills (time 3)

#### Child language

##### Vocabulary

Children completed the Expressive Vocabulary subtest from the Clinical Evaluation of Language Fundamentals (Semel, Wiig, & Secord, 2006). In this test children provide the name for a series of pictures of objects and actions of increasing difficulty (e.g. drawing, telescope). Maximum score is 54 (α = .84).

##### Sentence structure

Children completed the Receptive Grammar subtest of the Clinical Evaluation of Language Fundamentals (Semel et al., 2006). The child listens to a sentence read aloud by the examiner (e.g., “The bear is in the wagon”) and selects from a choice of four pictures the one that conveys its meaning. The sentences include a range of different syntactic structures. Maximum score is 26 (α = .83).

##### Sentence repetition

To assess expressive grammar, children repeated 20 sentences, 10 (five long/five short) containing transitive verbs and 10 (five long/five short) containing ditransitive verbs. The total number of sentences repeated correctly was recorded (α = .78).

#### Child reading/spelling

##### Word reading

Two subtests from the York Assessment of Reading for Comprehension measured single word reading (Snowling et al., 2009). In the Early Word Reading test, children read a list of 30 regular and irregular words found in early reading books (α = .98). For the Single Word Reading Test children read from a list of 60 words of increasing difficulty (α = .98). Raw scores were used for both measures. The measure of Single Word Reading was log-transformed to correct skew.

##### Spelling

Children spelled a list of five words (e.g., *cat, train*) dictated by the examiner, accompanied by pictures. Each response was marked globally and scored 0/1. Raw scores were used (α = .80).

### Socioeconomic status

#### Family SES

The educational level of both parents was assessed on a 6-point scale: 1 (*no formal qualifications*), 2 (*GCSEs* [exams taken at the end of compulsory education at age 16 in the United Kingdom] *or equivalent*), 3 (*A levels* [exams taken at the end of secondary education at ages 18–19 in the United Kingdom] *or equivalent*), 4 (*professional vocational qualification*), 5 (*undergraduate degree*), or 6 (*postgraduate degree*). In addition, the occupational status of both parents was assessed, using the Standard Occupational Classification (Office for National Statistics, 2010), which ranges from 1 (*unemployed*) to 10 (*managers, directors, senior officials*). Best occupational status was preferred to current occupational status, because many respondents were on parental leave from work at the time of data collection. A composite SES measure for use in analyses was derived by standardizing each of the four measures and taking the average of the *z* scores based on the total sample.

## Results

The means, standard deviations, and ranges for scores on all measures are provided in Table 1. There was between 0% and 24% data missing across variables with least data available on maternal skills (grammar, nonword repetition, and spoonerisms).

**Table 1.** Descriptive Statistics for the Measures of Maternal and Child Skills, as well as for Measures of the Home Literacy Environment.

		Distributions			
		N	M	SD	Range
Maternal measures—Time 1					
Maternal language					
1 Vocabulary		211	60.53	10.86	23–79
2 Grammar		192	11.16	4.43	0–20
3 Oral language		221	20.86	19.52	0–130
Maternal phonology					
4 Nonword repetition—ANRep		239	23.74	3.22	10–28
5 Nonword repetition—CTOPP		195	2.18	1.28	0–5
5 Spoonerism (time in seconds)		196	3.55	2.85	0.9–17.4
Home literacy environment—Time 2					
Storybook Exposure					
7 No. of children’s books		232	4.84	1.45	cat1–cat7
8 Frequency of bedtime reading		233	5.64	2.27	0–7/w
9 Frequency of reading at other times		233	4.04	2.80	0–17/w
10 Child title checklist		247	11.29	7.51	0–29
11 Child adult checklist		247	10.80	8.92	0–31
12 Adult title checklist		247	13.57	10.79	0–39
Direct literacy instruction					
13 Direct instruction of letters		233	3.70	1.32	cat1–cat5
14 Direct instruction of reading		233	3.17	1.68	cat1–cat5
15 Direct instruction of writing		233	3.19	1.38	cat1–cat5
Children’s measures—Time 3					
Child language					
16 Expressive vocabulary		232	26.06	9.96	2–47
17 Sentence structure		232	20.41	3.86	8–26
18 Sentence repetition		228	8.23	4.63	0–19
Child reading and spelling					
19 Early word recognition		232	15.86	8.93	0–30
20 Single word reading		230	9.90	9.82	0–44
21 Spelling		232	1.95	1.35	0–5
Socioeconomic status					
22 Maternal education		250	3.97	1.51	cat1–cat6
23 Maternal occupation		251	6.58	2.70	cat1–cat10
24 Paternal education		239	3.72	1.59	cat1–cat6
25 Paternal occupation		242	7.33	2.65	cat1–cat10

*Note*. Raw scores are presented in the table for all measures. ANRep = Adult Phonological Memory Test; CTOPP = Comprehensive Test of Phonological Processing.

Correlations between the variables assessing each construct were significant but varied in magnitude (Table 2). The intercorrelations among maternal language skills, maternal phonological skills, and storybook exposure measures were moderate. For children’s skills there were moderate intercorrelations among language measures and high correlations among measures of reading/spelling skills. Measures of direct instruction generally showed very low correlations with measures of indirect home literacy environment and maternal skills but correlations with child reading approached significance and were significant for spelling at Time 3.

**Table 2.** Correlations between observed variables.

		Correlations																			
		1	2	3	4	5	6	7	8	9	10	11	12	13	14	15	16	17	18	19	20
Maternal language—Time 1																					
1 Vocabulary																					
2 Grammar		.48**																			
3 Oral Language		.39**	.38**																		
Maternal phonology—Time 1																					
4 Nonword repetition—ANRep		.29**	.27**	.29**																	
5 Nonword repetition—CTOPP		.24**	.17*	.16*	.53**																
6 Spoonerism		−.39**	−.32**	−.30**	−.54**	−.44**															
HLE storybook exposure—Time 2																					
7 No. of children’s books		.29**	.09	.13	.14*	.11	−.16*														
8 Frequency of shared reading		.35**	.13	.29**	.05	.06	−.21**	.24**													
9 Mat. familiarity with children’s books		.60**	.41**	.35**	.24**	.20**	−.36**	.33**	.46**												
10 Mat. familiarity with adult fiction		.65**	.43**	.42**	.33**	.27**	−.41**	.21**	.38**	.68**											
HLE direct literacy instruction—Time 2																					
11 Direct literacy instruction		−.13	−.10	−.02	−.02	−.03	.01	.07	−.05	.10	−.08										
Children language—Time 3																					
12 Expressive vocabulary		.29**	.12	.17*	.15*	.05	−.19**	.17*	.27**	.24**	.25**	.02									
13 Sentence structure		.25**	.11	.10	.13*	−.01	−.18*	.16*	.28**	.20**	.18**	.05	.53**								
14 Sentence repetition		.36**	.15*	.16*	.24**	.17*	−.27**	.19**	.36**	.15*	.30**	−.02	.47**	.51**							
Children reading and spelling—Time 3																					
15 Early word recognition		.19**	.13	.11	.26**	.10	−.15*	.16*	.17*	.08	.15*	.12	.46**	.45**	.44**						
16 Single word reading		.18*	.15	.14	.16*	.04	−.17*	.05	.16*	.07	.16*	.06	.39**	.34**	.31**	.94**					
17 Spelling		.21**	.15*	.07	.27**	.13	−.22**	.22**	.19**	.10	.19**	.16*	.41**	.44**	.44**	.79**	.73**				
Socioeconomic status																					
18 Maternal education		.61**	.38**	.45**	.28**	.12	−.33**	.24**	.33**	.55**	.61**	−.03	.29**	.21**	.37**	.21**	.23**	.22**			
19 Maternal occupation		.42**	.19**	.40**	.20**	.21**	−.21**	.22**	.27**	.37**	.46**	−.02	.21**	.11	.21**	.15*	.14	.16*	.50**		
20 Paternal education		.47**	.24**	.25**	.16*	.09	−.21**	.18*	.14*	.31**	.51**	−.15*	.15*	.17*	.26**	.14*	.12	.16*	.53**	.42**	
21 Paternal occupation		.27**	.09	.21**	.15*	.14	−.10	.11	.08	.23**	.25**	−.09	.16*	.11	.21**	.21**	.07	.16*	.27**	.31**	.38**

*Note*. The variables used in the correlations are the same employed in the structural equation models. Coefficients in bold show correlations between observed variables within each latent variable. ANRep = Adult Phonological Memory Test; CTOPP = Comprehensive Test of Phonological Processing; HLE = home literacy environment.

*p < .05. **p < .01.

To test our hypotheses, structural equation models were constructed using Mplus 7.3 (Muthén & Muthén, 2015). Little’s Missing Completely at Random test showed that missing data were not missing completely at random; however, we proceeded to model the data initially with Full Information Maximum Likelihood estimators to allow for missing data. We subsequently reran the final model using listwise deletion to confirm that the pattern of findings did not change in any important ways.

**Figure 1.**
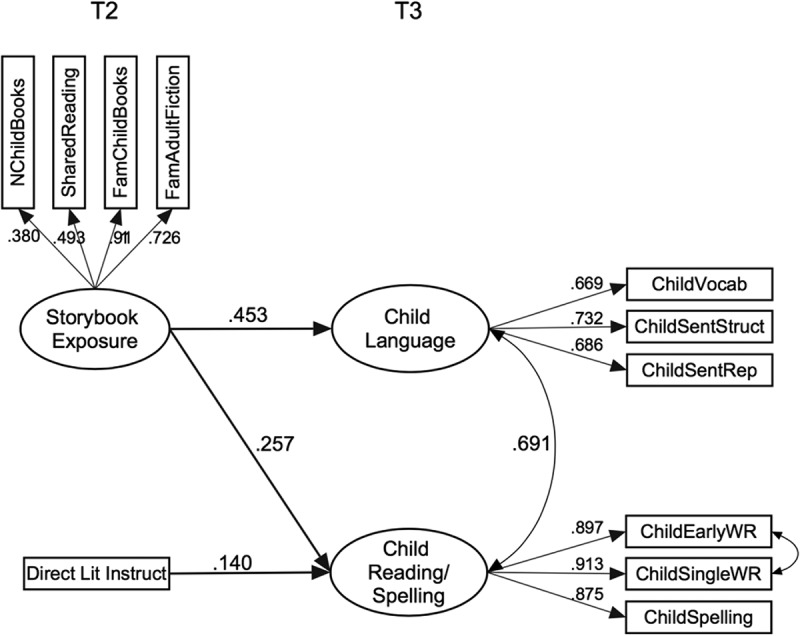
Model 1. Home literacy environment–child relationships.

Following confirmatory factor analysis to validate the constructs included at each time point, the first model uses data from measures of the home literacy environment (Time 2) and children’s outcomes (Time 3). We aimed to replicate previous findings showing that the home literacy environment predicts children’s language and reading/spelling skills. An initial saturated model was constructed with a correlation between the two measures of the home literacy environment (storybook exposure and direct literacy instruction) and with each of those measures as predictors of child language and child reading/spelling. The correlation between storybook exposure and direct literacy instruction was not significant, and dropping this path from the model resulted in no significant loss of fit, χ^2^ diff(1) = 0.352, *p *= .55. The simplified model is shown in Figure 1 and provides a good fit to the data: χ^2^(40) = 51.857, *p* = 0.099; root mean square error of approximation (RMSEA) = 0.035, 90% confidence interval (CI) [0.000, 0.060], standardized root mean square residual (SRMR) = 0.048, comparative fit index (CFI) = 0.989, Tucker–Lewis index (TLI) = 0.984. The proportion of variance explained (*R*^2^) for the main outcomes was .205 for child language and .085 for child reading/spelling skills. As expected, direct literacy instruction predicted child reading/spelling skills. Storybook exposure (i.e., number of children’s books, shared reading, parental familiarity with children books, and parental literacy instruction), on the other hand, predicted both children’s language and reading/spelling skills.

The second model assesses whether variations in the home literacy environment remain predictors of children’s language and reading/spelling skills after accounting for measures of maternal language and phonological skills. We began with a saturated model in which all relevant predictors of the child outcomes were included, and we dropped nonsignificant paths iteratively while observing changes in model fit. To rule out possible effects of SES on children’s language and reading/spelling skills, we added this variable as a covariate in the model and regressed it on the Time 2 and Time 3 variables (the appendix shows the initial model tested with nonsignificant paths shown with dashed lines). SES did not predict any of the outcomes. However, it is notable that SES had moderate to strong correlations with maternal measures at T1, particularly maternal language (model fit indices with significant and nonsignificant paths included), χ^2^(173) = 208.277, *p* = .035, RMSEA = 0.029, 90% CI [0.008, 0.042], SRMR = 0.051, CFI = 0.981, TLI = 0.977.

**Figure 2.**
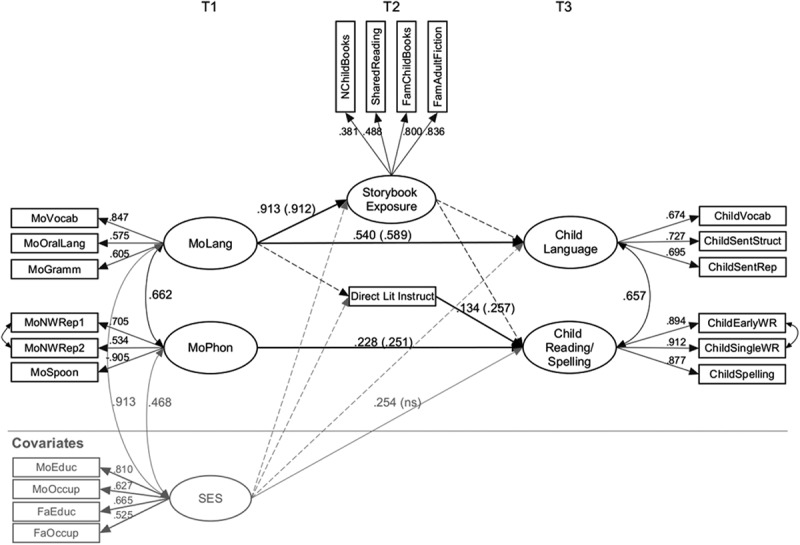
Model 2: Mother–home literacy environment–child relationships. *Note*. Structural equation path model showing the longitudinal relations between Time 1 measures of maternal language and phonology; Time 2 measures of home literacy environment; Time 3 measures of children’s language and reading/spelling skills. Ellipses indicate latent variables, and rectangles indicate observed variables. Values on single-headed arrows from the latent to the observed variables are standardized factor loadings, and values on single-headed arrows between the latent variables are standardized regression weights (values in parentheses stand for typically developing children only). Double-headed arrows indicate correlations.

The simplified model shown in Figure 2 is the final model with only significant paths retained. The model shows a good fit to the data, χ^2^(174) = 207.442, *p* = .042, RMSEA = 0.028, 90% CI [0.006, 0.041], SRMR = 0.051, CFI = 0.982, TLI = 0.979. As can be seen, neither of the paths from storybook exposure to child skills, (storybook exposure → child language; storybook exposure → child reading/spelling skills) were significant in this model. Dropping these nonsignificant paths resulted in no significant change to the fit of the model, χ^2^diff(1) = 0.292, *p* = .58. The only changes in the paths were that the moderate link between maternal language and child language became significant; SES now significantly predicted child reading/spelling. It is notable, however, that the path from direct literacy instruction → child reading/spelling skills remained significant after accounting for the effects of maternal phonological skills.

The proportion of variance explained (*R*^2^) for the latent outcomes is .834 for storybook exposure, .291 for child language, and .187 for child reading/spelling.

We had anticipated that there would be indirect effects from maternal language, via measures of the home literacy environment to the child language and literacy outcomes. However, none of the anticipated indirect effects from maternal language to child outcomes in this model were significant: maternal language → storybook exposure → child language, 95% CI of standardized indirect effect [−1.103, 0.496]; maternal language → storybook exposure → child reading/spelling skills, 95% CI of standardized indirect effect [−0.917, 0.307]; maternal language → direct literacy instruction → child reading/spelling skills, 95% CI of standardized indirect effect [−0.147, 0.223].

To confirm that the pattern of findings did not change in any important ways, we reestimated the final model using listwise deletion. Although this resulted in a major reduction in sample size (*N* = 112), the resulting model was essentially identical to the one that used full information maximum likelihood, except that the path from SES to child reading/spelling fell to below significance (standardized regression coefficient = .19) and that from direct literacy instruction was slightly stronger (standardized regression coefficient = .20). In short, the pattern from the model using the full data set, and that from a model in which listwise deletion, is used show essentially equivalent patterns.

The preceding analyses were conducted pooling data across typically developing and at-risk children. However, we know from previous research that the relationship between the home literacy environment and children’s language and literacy skills may differ in families of children with and without language impairment (Skibbe et al., 2008). To make sure the high percentage of language impaired children did not unduly influence the results, we reran the final model just described with the typically developing children only (*N* = 162). The pattern of results remained essentially the same, with a good fit to the data, χ^2^(174) = 188.571, *p* = .213, RMSEA = 0.023, 90% CI [0.000, 0.043], SRMR = 0.055, CFI = 0.987, TLI = 0.985. The resulting model shown in Figure 2 indicates in parentheses the standardised regression weights for the typically developing sample. The only difference between this model and that using data from the whole sample is that the path from SES to child reading/spelling is not significant (standardized regression coefficient = .138). The proportion of variance explained (*R*^2^) for the latent outcomes is .831 for storybook exposure, .346 for child language, and .170 for child reading/spelling. We can therefore conclude that the model provides an adequate fit to the data for both the full sample including language impaired children and for typically developing children also.

## Discussion

This study explored the role of the home literacy environment as a predictor of children’s language and reading skills 1 year after school entry, after accounting for the effects of maternal language and phonological abilities. As expected, maternal language skills were a significant predictor of storybook exposure but not direct literacy instruction. In addition, storybook exposure predicted children’s general language and reading/spelling skills, whereas direct literacy instruction predicted only children’s reading/spelling skills. This pattern of effects, ignoring possible effects from mothers’ own language and phonological skills, are broadly in line with previous findings (Bus et al., 1995; Hamilton et al., 2016). However, the most striking result here is that once mothers’ language and phonological skills are taken into account, storybook exposure is no longer a predictor of children’s language or reading/spelling skills. In contrast, direct literacy instruction does remain a predictor, albeit a weak one, of children’s reading/spelling skills after accounting for variations in maternal language and phonological abilities. We speculate that its influence on reading and spelling may have been stronger had outcomes been measured at an earlier stage in formal reading instruction.

Our findings suggest that the informal home literacy environment does not directly influence children’s language and reading development. A parsimonious explanation for our findings is that the effects of the informal home literacy environment reflect genetic influences, that is, mothers with good language skills pass on genes that confer good language skills. However, in a design such as that used here, it is impossible to separate genetic influences from those attributable to *ge* correlation. Moreover, it is notable that the measures of storybook exposure correlated highly with both maternal language and SES, suggesting that maternal education rather than maternal genes is also a plausible driver of the effects.

In contrast, and perhaps not surprising, the effects of direct literacy instruction in the home on children’s early mastery of the mechanics of reading and spelling do appear to reflect environmental influences, although the influence is weak (only about 1.7% of the variance in children’s literacy skills is explained by this measure). The predictive role of parental teaching of literacy on children’s reading/spelling skills at or around school entry has been reported in numerous studies from various countries (Evans et al., 2000; Foy & Mann, 2003; Manolitsis et al., 2013; Martini & Sénéchal, 2012). This “formal” aspect of home literacy has also been reported to be independent of informal home-based literacy activities (Phillips & Lonigan, 2009; Sénéchal & LeFevre, 2002). Our findings align with this body of research and suggest that the extent to which parents teach their young children about letters and words is independent of maternal language and phonological skills. In other words, mothers with better language and phonological skills are no more likely to engage in activities to teach children to decode print than mothers with less well-developed language skills. Consistent with this are suggestions that formal literacy activities in the home may be influenced not by parents’ skills but by their values and beliefs (Martini & Sénéchal, 2012).

As anticipated, measures of maternal language did predict variations in the amount of storybook exposure, explaining more than 80% of the variance in this latent variable (even after controlling for aspects such as SES). However, it is striking that maternal language skills alone accounted for so much variance in the informal home literacy environment. Some of the measures of storybook exposure used in this study clearly depend on mothers’ own language and reading abilities (identification of book authors and book titles). However, others quantify different facets of home literacy practices (number of children’s books and shared reading) and are expected to reflect the frequency of informal literacy activities. It is important to highlight, however, that the measures of storybook exposure used in this study do not reflect the *quality* of parent–child interactions around storybooks. Measures of the quality of home literacy interactions, such as the amount and type of parental extratextual talk around storybooks, have been shown to predict children’s language and early literacy skills (Deckner, Adamson, & Bakeman, 2006; Hindman, Skibbe, & Foster, 2014), and such measures may be more likely to mediate the relationship between parental language and child skills than the measures used in the current study. In other words, parents with better language skills themselves may provide richer, more interactive experiences with print for their children (e.g., by using decontextualized language, prompting children to explain/predict events in the story), which in turn may scaffold children’s developing language skills.

If we leave aside the effects of mothers’ own skills, we found that direct instruction in the home predicted child reading/spelling skills, whereas storybook exposure influenced child language, in line with the model of Senechal and Lefevre (2002). We also found that storybook exposure had an impact on child reading/spelling skills, which was not originally predicted by Senechal and Lefevre (2002) but is reported in previous research (Bus et al., 1995; Kim, 2009). Notwithstanding this, we found that the relationship between the informal home literacy environment and child language and literacy was largely explained by variance that was shared with maternal skills, consistent with Van Bergen et al. (2016). It therefore seems possible that such apparently “environmental” effects reflect at least in part the role of heritable influences. This study had a number of limitations. We note that the measures of home literacy environment do not include direct observations in the home or measures of the quality of interactions around literacy. Further, our models include measures of only mothers’ and not fathers’ skills, whereas our measure of SES is based on data from mothers and fathers. Although there were missing data and parents with lower literacy scores in particular completed fewer tasks, this is often an inevitable feature of research with families in whom some parents have neurodevelopmental disorders; arguably the replication of the findings across different models provides some validation; however, further replication in an independent sample is desirable.

In summary, our findings suggest that it is not solely the amount of literacy activity a child is exposed to that determines his or her early language and literacy development; it is also the linguistic ability of the parent (in this case the mother) who is providing the literacy environment at home. It follows that much of what has traditionally been attributed to the home literacy environment may be a proxy for parental skills. It is important, however, to emphasise that our findings do not speak to the possible benefits of interventions to improve children’s language and reading skills. Indeed if, as we have speculated, the relationship between the informal home literacy environment and child language and reading/spelling outcomes reflects the effects of *ge* correlations, this does not mean that interventions to improve or enrich the home literacy environment will not be effective in promoting children’s language and reading development; such efforts need to be continued to improve the outcomes of children with poor levels of language and limited opportunities for literacy.

## Conclusions

This is the first study to quantify the amount of variance in the home literacy environment explained by maternal language skills. We showed, similar to other studies, that children’s language and reading/spelling skills are related to storybook exposure, but we propose that this could be interpreted as a proxy for genetic effects or *ge* correlations. Direct literacy instruction, however, is not influenced by parents’ skills and might represent a true environmental effect on children’s reading and spelling.

## Figures and Tables

**Figure:**
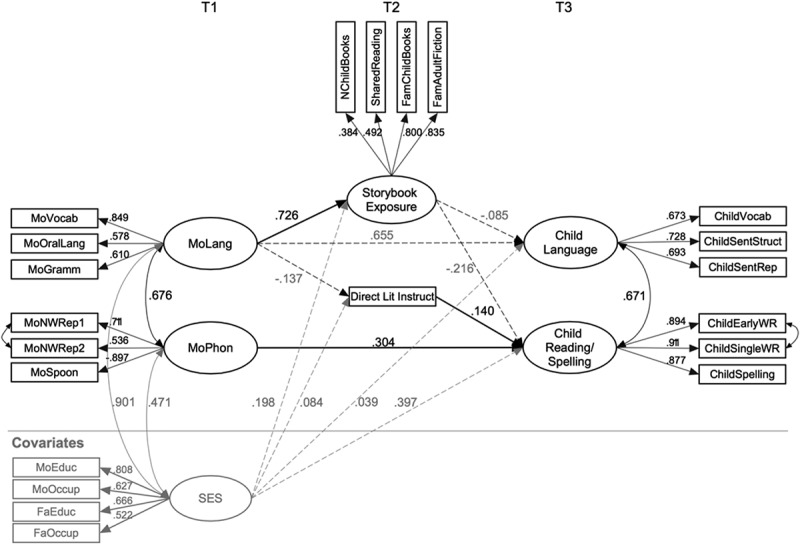
*Note*. Structural equation path model showing the longitudinal relations between Time 1 measures of maternal language and phonology; Time 2 measures of home literacy environment; Time 3 measures of children’s language and reading/spelling skills. Ellipses indicate latent variables, and rectangles indicate observed variables. Values on single-headed arrows from the latent to the observed variables are standardized factor loadings, and values on single-headed arrows between the latent variables are standardized regression weights (dashed lines represent nonsignificant paths). Double-headed arrows indicate correlations.

## Appendix

MODEL 2 with significant and nonsignificant paths: Mother–HLE–Child relationships
